# Supplementary material for: Impact of whole-genome duplications on structural variant evolution in Cochlearia
Source: Nat Commun. 2024 Jun 25;15:5377. doi: 10.1038/s41467-024-49679-y (PMC11199601; doi:10.1038/s41467-024-49679-y)
Supplement: Supplementary file 1 — Supplementary Information [file 41467_2024_49679_MOESM1_ESM.pdf]

Impact of whole-genome duplications on structural variant evolution in *Cochlearia*

Hämälä *et al.*

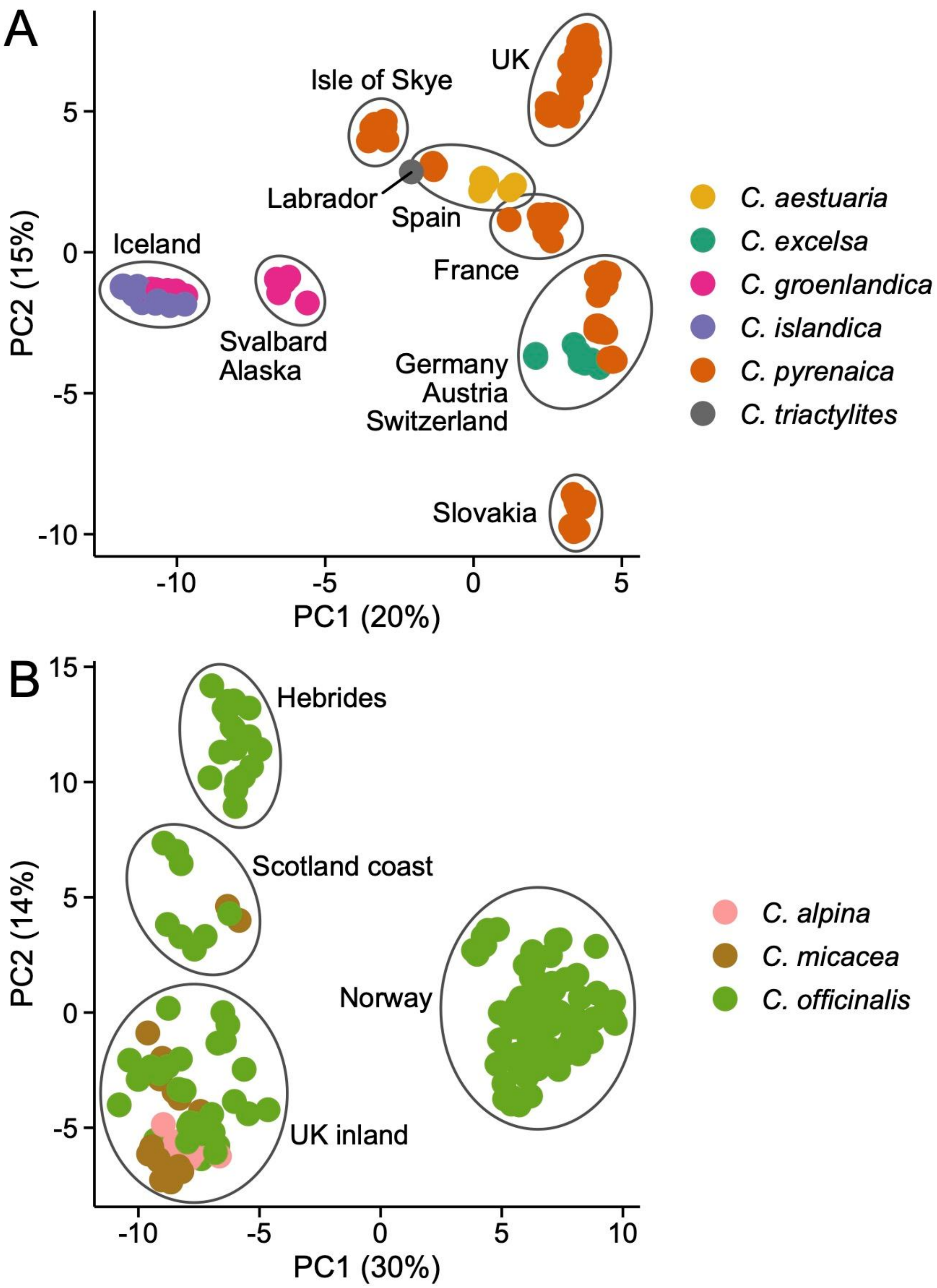

Supplementary Figure 1. PCA conducted separately on diploids (A) and tetraploids (B). Species are marked with colours. The proportion of variance explained by the PCs is shown in parentheses. Source data are provided as a Source Data file.

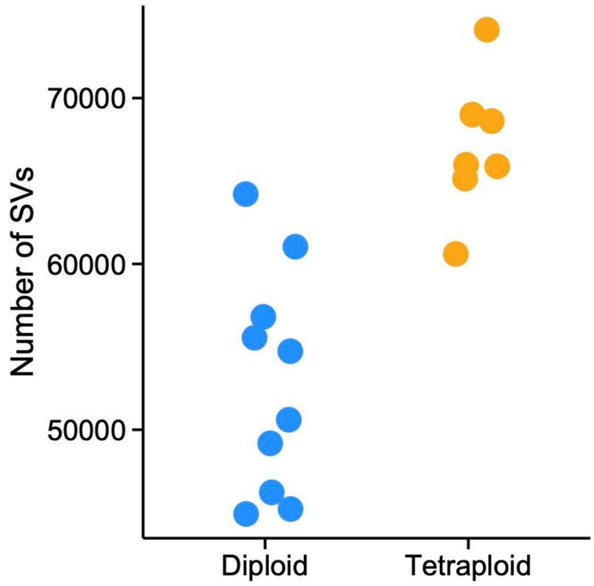

Supplementary Figure 2. Number of SVs found in long-read sequenced samples after equalising the alignments to same number of base pairs covered (mean read length × number of reads). Poisson model-based LRT for a difference between the ploidies: deviance = 13980, DF = 1, $P < 2 \times 10^{-16}$. Source data are provided as a Source Data file.

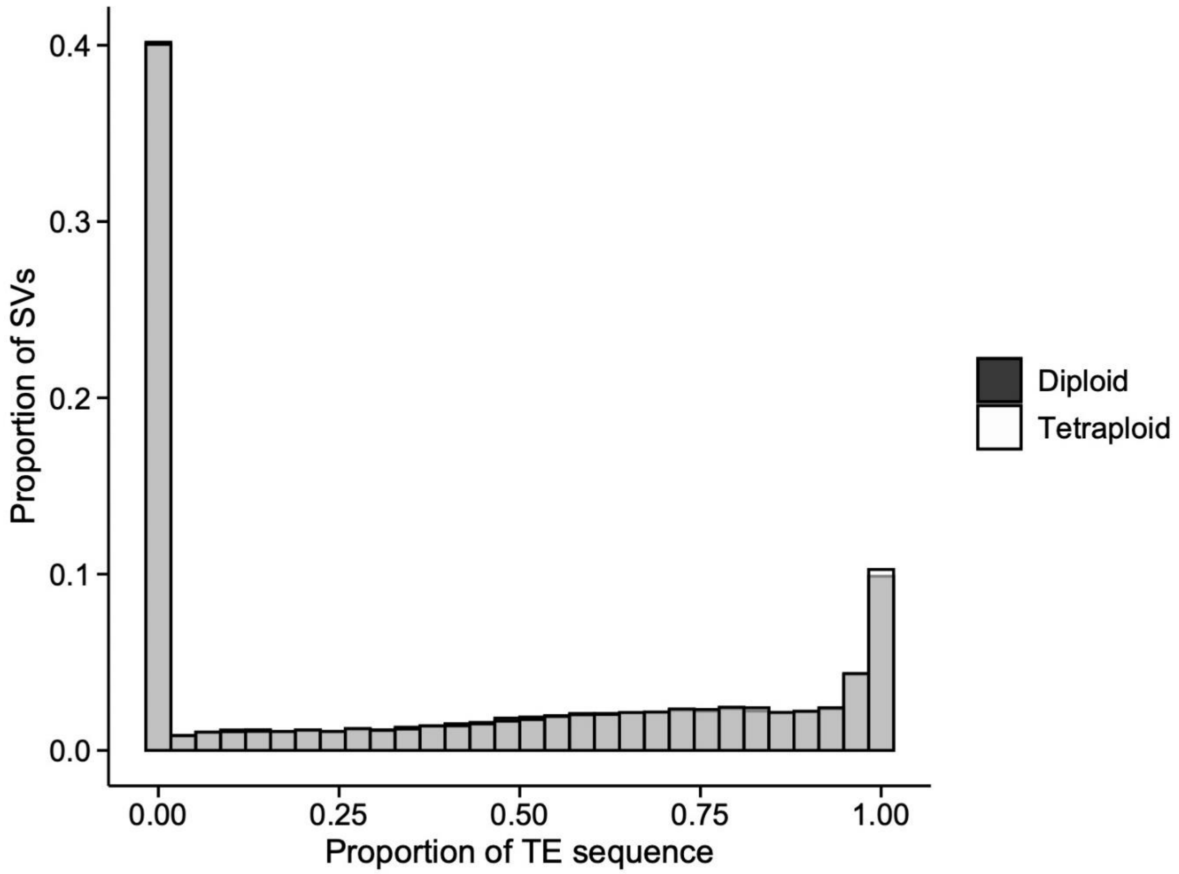

Supplementary Figure 3. Proportion of TE sequence found in SVs. Source data are provided as a Source Data file.

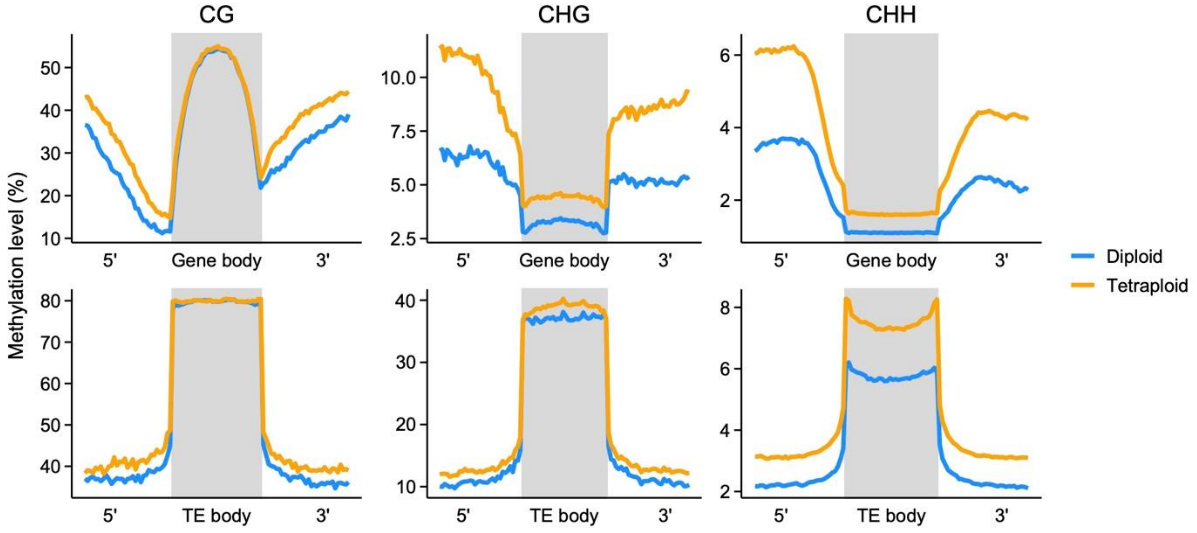

Supplementary Figure 4. Methylation levels across meta-genes and meta-TEs, shown for CG, CHG, and CHH contexts. Note the difference in *y*-axis scales between the panels. Source data are provided as a Source Data file.

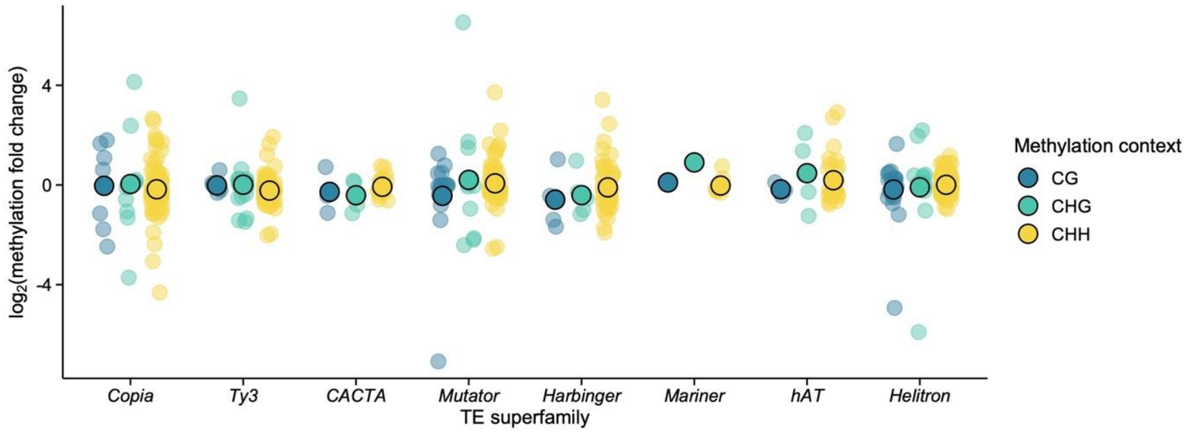

Supplementary Figure 5. Methylation difference between diploids and tetraploids shown for differentially methylated TE families. Source data are provided as a Source Data file.

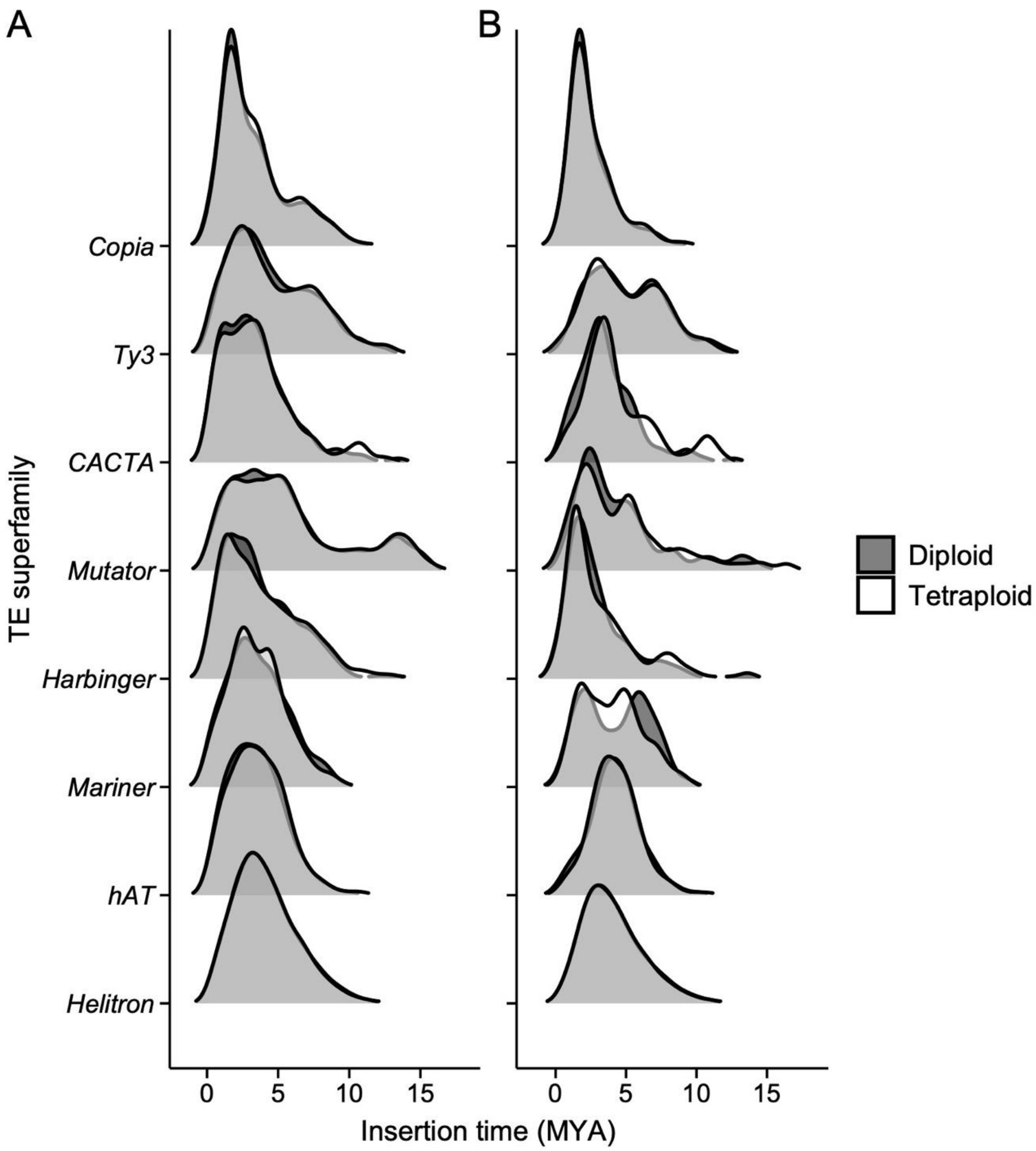

Supplementary Figure 6. Distributions of TE insertions times in diploids and tetraploids. A: All TE families. **B**: Differentially methylated TE families. Source data are provided as a Source Data file.

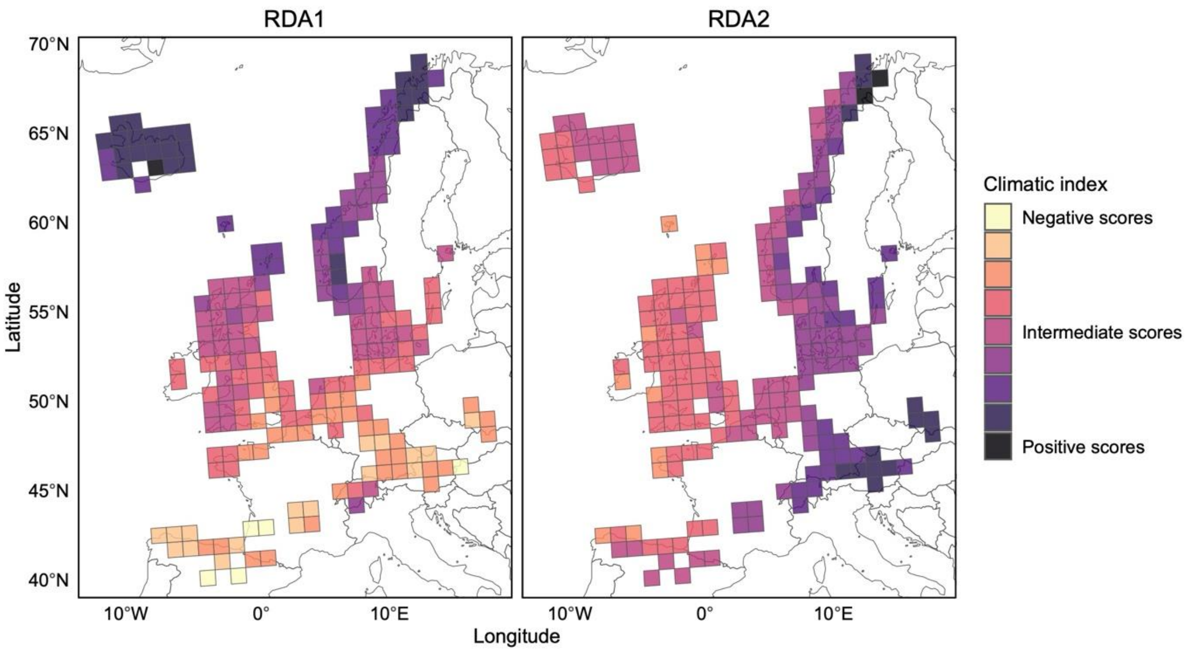

Supplementary Figure 7. Climatic index (based on combined SV and SNP outliers) predicted across the European range of our focal *Cochlearia* species. Similar colours indicate higher similarity in the genetic composition of the populations. Shown are two RDA axes used in the prediction of climatic distance between SVs and SNPs. Map data: GISCO, licensed under CC by 4.0. Source data are provided as a Source Data file.

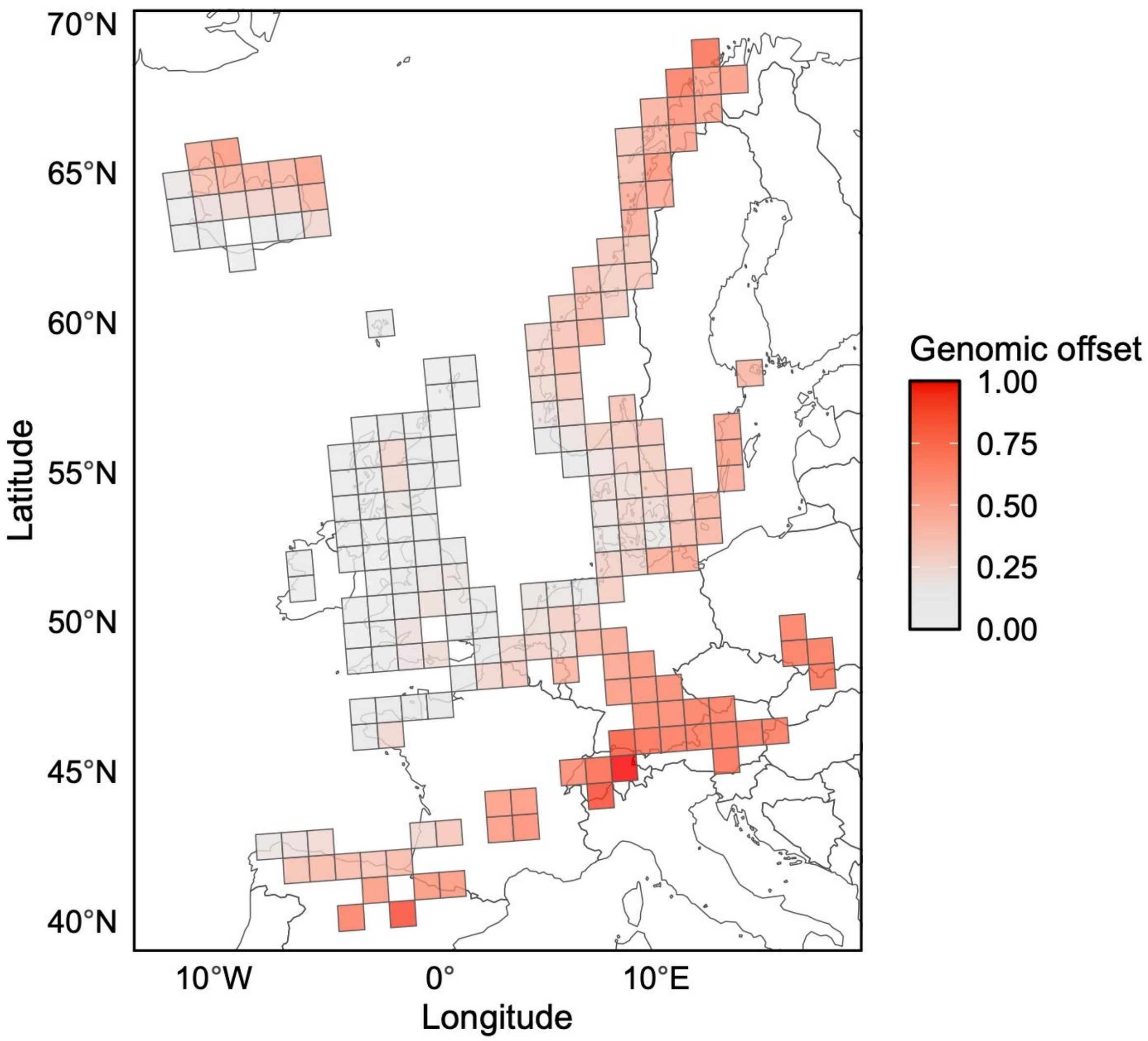

Supplementary Figure 8. Genomic offset between past (1970 – 2000) and future (2061 – 2080) climatic conditions, predicted using all climate-associated variants (SVs and SNPs). Colour scale indicates the relative level of genetic change that would be required to track climate change. Map data: GISCO, licensed under CC by 4.0. Source data are provided as a Source Data file.

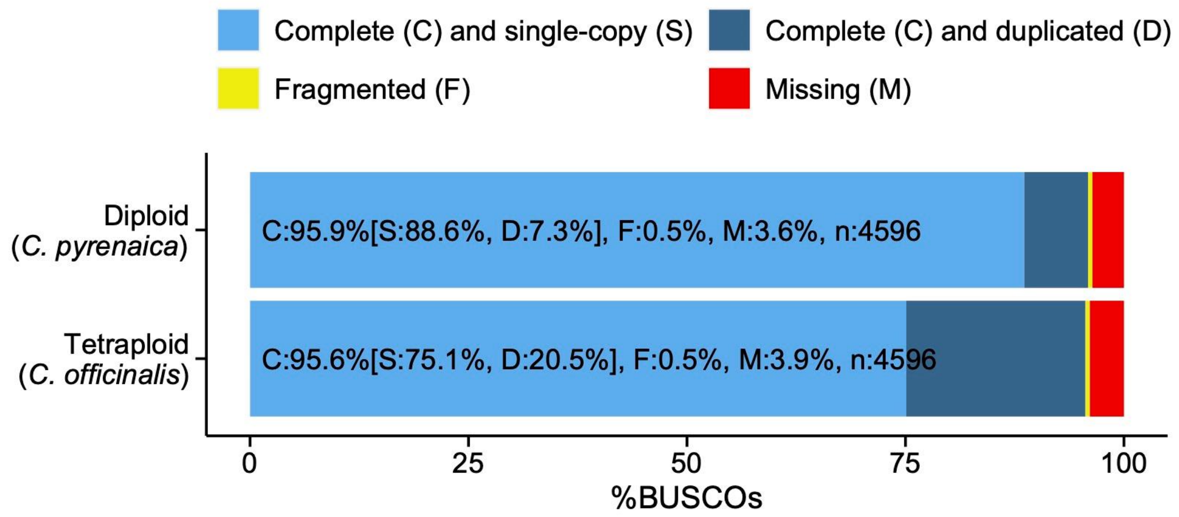

Supplementary Figure 9. BUSCO results in the newly assembled diploid and tetraploid genomes. Source data are provided as a Source Data file.

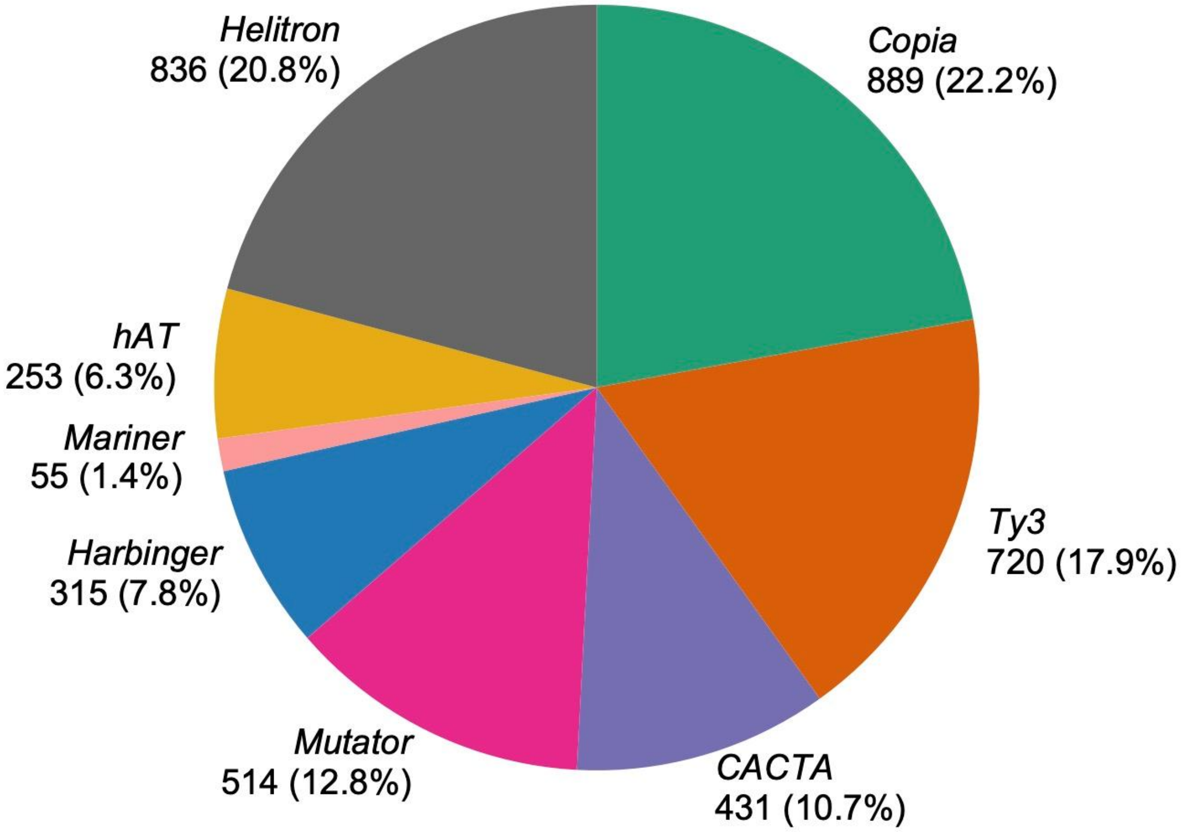

Supplementary Figure 10. Number and proportion of TE superfamilies annotated in three different *Cochlearia* assemblies. Source data are provided as a Source Data file.

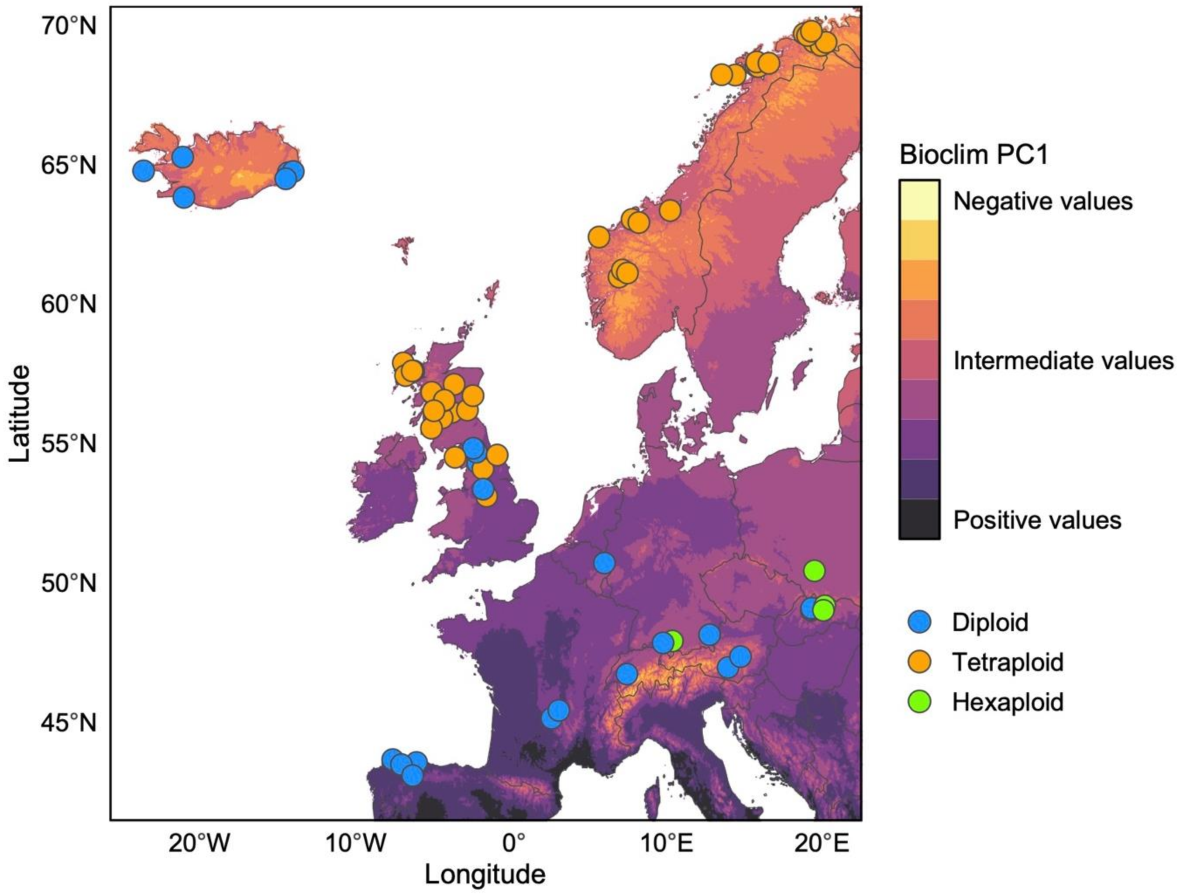

Supplementary Figure 11. Geographical variation across 11 bioclimatic variables used in our GEA, summarised as the first principal component (PC) of the data. Coloured circles show 70 *Cochlearia* populations used in the analyses. Source data are provided as a Source Data file. Following bioclim variables were used (in the order of their significance in explaining genetic variation among the populations):

bio2 = Mean diurnal range
bio3 = Isothermality
bio9 = Mean temperature of driest quarter
bio15 = Precipitation seasonality
bio18 = Precipitation of warmest quarter
bio8 = Mean temperature of wettest quarter
bio10 = Mean temperature of warmest quarter
bio7 = Temperature annual range
bio1 = Annual mean temperature
bio5 = Max temperature of warmest month
bio11 = Mean temperature of coldest quarter

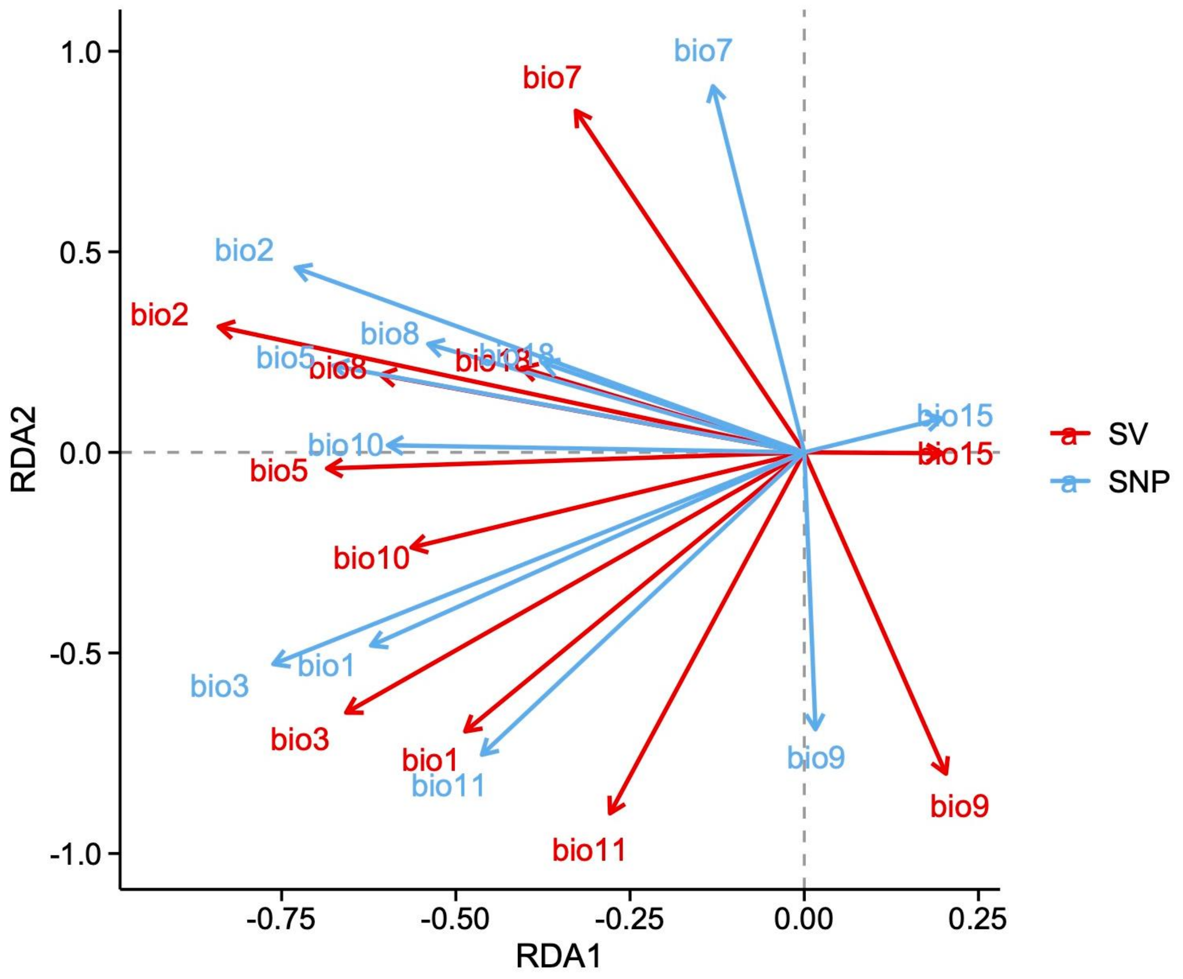

Supplementary Figure 12. Loadings of 11 bioclimatic variables on first two RDA axes, shown for outlier SVs and SNPs. Length of the arrows indicate the load of each variable on the axes. Source data are provided as a Source Data file.

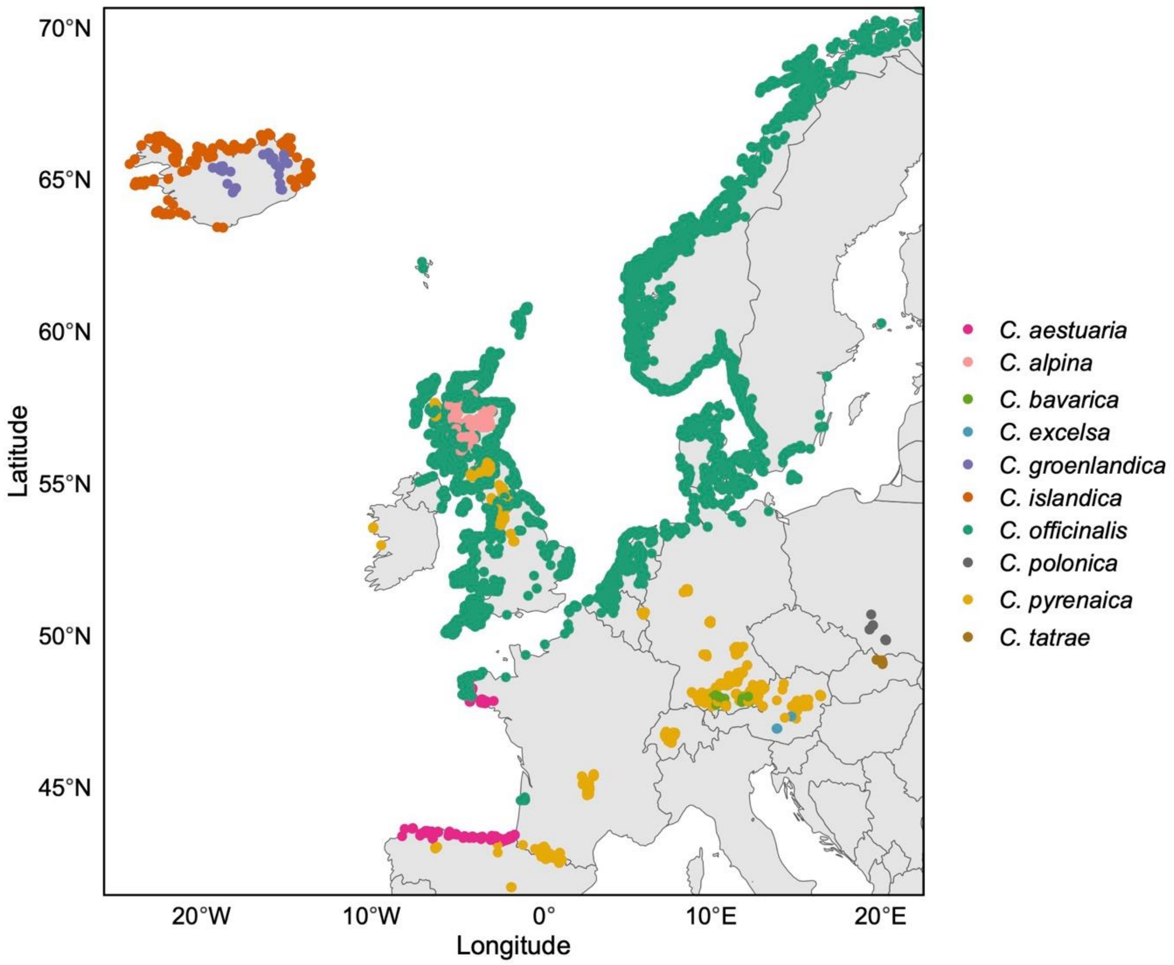

Supplementary Figure 13. Occurrence records for the *Cochlearia* species acquired from GBIF (https://www.gbif.org) and manually curated. Source data are provided as a Source Data file.

Supplementary Table 1. Summary of the long-read sequenced samples.

ID	Ploidy	*Cochlearia* species	Country	Sequencing technology	Read length N50 (kb)	Mean depth	Mapping rate (%)
C10	2	*pyrenaica*	OS	ONT	8.9	26.1	98
C12	2	*pyrenaica*	DE	ONT	5.8	24.1	99
C13*	2	*pyrenaica*	DE	ONT	8.7	9.0	98
C14	2	*aestuaria*	ES	ONT	11.8	24.6	98
C17	2	*pyrenaica*	FR	ONT	7.6	24.0	97
C18*	2	*groenlandica*	IS	ONT	4.3	9.1	97
C19	2	*aestuaria*	ES	ONT	10.4	28.4	99
C2	2	*excelsa*	OS	ONT	7.8	28.9	98
C20	2	*pyrenaica*	OS	ONT	6.8	14.7	99
C21	2	*pyrenaica*	FR	ONT	6.4	25.3	97
C23*	2	*pyrenaica*	UK	ONT	11.4	6.3	99
C5*	2	*islandica*	IS	ONT	10.3	8.2	96
LAB 22	2	*pyrenaica*	UK	ONT	7.0	62.5	98
NEN 30	2	*pyrenaica*	UK	PacBio	16.1	21.9	100
BPS 1	4	*officinalis*	UK	ONT	7.3	82.6	100
CUR	4	*officinalis*	UK	ONT	5.4	48.7	92
ELI 23	4	*officinalis*	UK	ONT	7.2	75.8	98
ONI 12	4	*officinalis*	UK	ONT	7.6	58.1	93
ROT 20	4	*officinalis*	UK	ONT	7.4	85.2	99
ROT 26	4	*officinalis*	UK	PacBio	7.4	57.3	99
TRE 13	4	*officinalis*	UK	ONT	8.5	69.4	95
C27	6	*tatrae*	SL	ONT	5.8	18.3	97
C24	8	*anglica*	UK	ONT	4.9	27.6	98

*Excluded from the main analyses due to low sequencing depth.

Supplementary Table 2. Validation of Sniffles2 (v2.2) using simulated autotetraploids.

Genomic feature	Allelic dosage	Simulated depth									
		5×		10×		20×		40×		80×	
		R	P	R	P	R	P	R	P	R	P
All	1	0.25	0.99	0.50	0.99	0.76	0.98	0.89	0.98	0.97	0.98
	2	0.48	0.98	0.75	0.98	0.91	0.96	0.96	0.96	0.98	0.94
	3	0.64	0.98	0.85	0.97	0.95	0.94	0.97	0.93	0.98	0.92
	4	0.70	0.98	0.86	0.96	0.93	0.92	0.96	0.90	0.98	0.89
Exon	1	0.26	1.00	0.52	1.00	0.79	0.99	0.92	1.00	0.98	0.99
	2	0.51	1.00	0.79	0.99	0.93	0.99	0.98	0.99	0.99	0.99
	3	0.67	0.99	0.88	0.99	0.97	0.99	0.99	0.99	1.00	0.99
	4	0.72	0.99	0.89	0.99	0.95	0.99	0.98	0.99	0.99	0.98
Intron	1	0.26	1.00	0.52	1.00	0.79	0.99	0.91	0.99	0.98	0.99
	2	0.51	1.00	0.78	0.99	0.93	0.99	0.98	0.99	0.99	0.99
	3	0.67	0.99	0.87	0.99	0.97	0.99	0.99	0.98	0.99	0.98
	4	0.72	1.00	0.88	0.99	0.95	0.99	0.98	0.98	0.99	0.98
1 kb up- and downstream	1	0.26	1.00	0.52	1.00	0.79	0.99	0.92	0.99	0.98	0.99
	2	0.51	0.99	0.79	0.99	0.94	0.99	0.98	0.99	0.99	0.99
	3	0.67	0.99	0.88	0.99	0.97	0.99	0.99	0.99	0.99	0.98
	4	0.73	1.00	0.89	0.99	0.95	0.99	0.98	0.98	0.99	0.98
Intergenic (> 1 kb away from genes)	1	0.18	0.59	0.38	0.63	0.64	0.63	0.80	0.61	0.82	0.60
	2	0.37	0.64	0.62	0.60	0.81	0.60	0.87	0.68	0.92	0.57
	3	0.51	0.64	0.74	0.61	0.88	0.57	0.91	0.55	0.94	0.54
	4	0.59	0.66	0.76	0.61	0.86	0.55	0.90	0.51	0.93	0.50

Shown are recall (R) and precision (P) estimates for different levels of read depth over simulated insertions and deletions found overlapping different genomic features.

Supplementary Table 3. **Validation of vg (v1.53.0) using simulated autotetraploids.**

Allelic dosage	Simulated depth									
	5×		10×		20×		40×		80×	
	R	P	R	P	R	P	R	P	R	P
1	0.42	1.00	0.67	1.00	0.81	0.99	0.85	0.99	0.86	0.99
2	0.67	1.00	0.81	1.00	0.85	1.00	0.86	0.99	0.86	1.00
3	0.76	1.00	0.84	1.00	0.86	1.00	0.86	1.00	0.86	1.00
4	0.79	1.00	0.85	0.99	0.86	1.00	0.86	1.00	0.86	1.00

Shown are recall (R) and precision (P) estimates for different levels of read depth over simulated insertions and deletions.

Supplementary Table 4. Results from GO enrichment analyses conducted on GEA outliers.

GO term	Dataset	Fold enrichment	*P*-value
Maintenance of seed dormancy	Diploid	33	0.0015
Cell surface receptor signaling pathway	Diploid	18	0.0054
Plant-type hypersensitive response	Tetraploid	29	0.0022
Starch catabolic process	Tetraploid	22	0.0031
Formaldehyde catabolic process	Tetraploid	22	0.0031
Defense response to bacterium	Tetraploid	14	0.0078
Oligopeptide transport	All	21	0.0003
Leaf development	All	3	0.0014
Xanthophyll metabolic process	All	25	0.0024
Formaldehyde catabolic process	All	22	0.0033
Ethylene biosynthetic process	All	13	0.0099

Shown are GO terms with $P < 0.01$, one-sided Fisher's exact test.

Supplementary Table 5. Species used in estimating coding sequence conservation with GERP++.

Species	Source
Aethionema arabicum	https://plantcode.cup.uni-freiburg.de/aetar_db/
Arabidopsis thaliana	Ensembl Plants
Arabis alpina	http://www.arabis-alpina.org/refseq.html
Boechera stricta	Phytozome
Brassica rapa	Ensembl Plants
Camelina sativa	Ensembl Plants
Capsella rubella	Phytozome
Cardamine hirsuta	http://chi.mpipz.mpg.de/assembly.html
Carica papaya	Phytozome
Citrus clementina	Ensembl Plants
Cucumis sativus	Ensembl Plants
Draba nivalis	https://doi.org/10.5061/dryad.pg4f4qrm4
Eucalyptus grandis	Ensembl Plants
Eutrema salsugineum	Phytozome
Fragaria vesca	Phytozome
Glycine max	Ensembl Plants
Gossypium raimondii	Ensembl Plants
Juglans regia	Ensembl Plants
Lobularia maritima	https://ngdc.cncb.ac.cn/gwh/Assembly/9796/show
Malus domestica	Ensembl Plants
Manihot esculenta	Ensembl Plants
Medicago truncatula	Ensembl Plants
Pistacia vera	Ensembl Plants
Populus trichocarpa	Ensembl Plants
Quercus lobata	Ensembl Plants
Raphanus sativus	https://plantgarden.jp/
Schrenkiella parvula	Phytozome
Theobroma cacao	Ensembl Plants
Vitis vinifera	Ensembl Plants
