# Supplementary material for: Impact of whole-genome duplications on structural variant evolution in Cochlearia
Source: Nat Commun. 2024 Jun 25;15:5377. doi: 10.1038/s41467-024-49679-y (PMC11199601; doi:10.1038/s41467-024-49679-y)
Supplement: Supplementary file 3 — Reporting Summary [file 41467_2024_49679_MOESM3_ESM.pdf]

Corresponding author(s): Levi Yant
Last updated by author(s): 07/06/2024

Reporting Summary

Nature Portfolio wishes to improve the reproducibility of the work that we publish. This form provides structure for consistency and transparency in reporting. For further information on Nature Portfolio policies, see our Editorial Policies and the Editorial Policy Checklist.

Statistics

For all statistical analyses, confirm that the following items are present in the figure legend, table legend, main text, or Methods section.

n/a	Confirmed	
☐	☒	The exact sample size (*n*) for each experimental group/condition, given as a discrete number and unit of measurement
☐	☒	A statement on whether measurements were taken from distinct samples or whether the same sample was measured repeatedly
☐	☒	The statistical test(s) used AND whether they are one- or two-sided<br>*Only common tests should be described solely by name; describe more complex techniques in the Methods section.*
☐	☒	A description of all covariates tested
☐	☒	A description of any assumptions or corrections, such as tests of normality and adjustment for multiple comparisons
☐	☒	A full description of the statistical parameters including central tendency (e.g. means) or other basic estimates (e.g. regression coefficient) AND variation (e.g. standard deviation) or associated estimates of uncertainty (e.g. confidence intervals)
☐	☒	For null hypothesis testing, the test statistic (e.g. *F*, *t*, *r*) with confidence intervals, effect sizes, degrees of freedom and *P* value noted<br>*Give P values as exact values whenever suitable.*
☐	☒	For Bayesian analysis, information on the choice of priors and Markov chain Monte Carlo settings
☐	☒	For hierarchical and complex designs, identification of the appropriate level for tests and full reporting of outcomes
☐	☒	Estimates of effect sizes (e.g. Cohen's *d*, Pearson's *r*), indicating how they were calculated

Our web collection on statistics for biologists contains articles on many of the points above.

Software and code

Policy information about availability of computer code

Data collection	Guppy v6.1.5, Deepsignal-plant v0.1.6
Data analysis	hifiasm v0.16.1-r375, purge_dups v1.2.5, BUSCO v5.3.2, EDTA v2.0.0, BLAST v2.12.0+, Trimmomatic v0.38, bwa-mem2 v2.0pre2, GATK v4.4.0, vegan v2.6-4, minimap2 v2.24-r1122, Sniffles v2.2, NanoPlot v1.32.1, SURVIVOR v1.0.6, PBSIM v2.0.2, Updog v2.1.3, qvalue v2.32.0, TELR v1.0, MAFFT v7.490, Phangorn v2.11.1, GERP++ v2.1, vg v1.45.0, BCFtools v1.9, mason v2.0.9, BayPass v2.31, SweepFinder2 v.1.0, TopGO v2.52.0, R v4.3.0, CoordinateCleaner v3.0, custom scripts available at https://github.com/thamala/polySV

For manuscripts utilizing custom algorithms or software that are central to the research but not yet described in published literature, software must be made available to editors and reviewers. We strongly encourage code deposition in a community repository (e.g. GitHub). See the Nature Portfolio guidelines for submitting code & software for further information.

Data

Policy information about availability of data

All manuscripts must include a data availability statement. This statement should provide the following information, where applicable:

- Accession codes, unique identifiers, or web links for publicly available datasets
- A description of any restrictions on data availability
- For clinical datasets or third party data, please ensure that the statement adheres to our policy

Sequence data for this study have been deposited in the European Nucleotide Archive (ENA) at EMBL-EBI under accession number PRJEB66308 (https://www.ebi.ac.uk/ena/browser/view/PRJEB66308). Scripts for conducting the analyses are available at https://github.com/thamala/polySV

Research involving human participants, their data, or biological material

Policy information about studies with human participants or human data. See also policy information about sex, gender (identity/presentation), and sexual orientation and race, ethnicity and racism.

Reporting on sex and gender	N/A
Reporting on race, ethnicity, or other socially relevant groupings	N/A
Population characteristics	N/A
Recruitment	N/A
Ethics oversight	N/A

Note that full information on the approval of the study protocol must also be provided in the manuscript.

Field-specific reporting

Please select the one below that is the best fit for your research. If you are not sure, read the appropriate sections before making your selection.

☐ Life sciences ☐ Behavioural & social sciences ☒ Ecological, evolutionary & environmental sciences

For a reference copy of the document with all sections, see nature.com/documents/nr-reporting-summary-flat.pdf

Ecological, evolutionary & environmental sciences study design

All studies must disclose on these points even when the disclosure is negative.

Study description	Population level study in a hierarchical design (population replicates within known lineages)
Research sample	Individual samples of Cochlearia spp representing the widest available cytotype, species, and population diversity, given available herbaria- or fied-based sampling.
Sampling strategy	The sample size reflects the availability of herbaria collections and natural populations. Within each population we sampled plants a minimum of 2 meters apart and aimed to sample at least 10 plants, given that population sizes were large in order to not impact the natural populations.
Data collection	Data collection was by DNA sequencing as described in the manuscript.
Timing and spatial scale	Samples were obtained from herbaria, with ultimate sourcing of the material into the herbaria of approximately 19 years (beginning in 2004) until 2022. Spatial scale represents the natural range of Cochlearia spp (Austria, Belgium, England, France, Germany, Iceland, Norway, Scotland, Slovakia, Spain, Switzerland).
Data exclusions	From the sequencing dataset none of the listed individuals were excluded.
Reproducibility	There were no attempts to reproduce the experiment due to its large scale and logistical demands.
Randomization	Field samples were collected along transects of the natural populations in the field.
Blinding	No blinding has been applied.

Did the study involve field work? ☒ Yes ☐ No

Field work, collection and transport

Field conditions	All sampled populations were natural. All populations grew in similar vegetation (rocky outcrops on the coasts or in grasslands).
Location	Locations are listed in Dataset_S1
Access & import/export	The samples were collected in compliance with local, national, and international laws in the following countries: Austria, Belgium, England, France, Germany, Iceland, Norway, Scotland, Slovakia, Spain, Switzerland. Material from collections under curation/ international exchange of Heidelberg botanical collections and herbarium (acronym HEID). One collection required a permit, which was gained by the Slovak Ministry for Environment, permission No 062-219/18. Where applicable and relevant we received permission from Nagoya focal points in each country and submitted the a Due Diligence Declaration to the Competent Authority.
Disturbance	No disturbances were caused by the study.

Reporting for specific materials, systems and methods

We require information from authors about some types of materials, experimental systems and methods used in many studies. Here, indicate whether each material, system or method listed is relevant to your study. If you are not sure if a list item applies to your research, read the appropriate section before selecting a response.

Materials & experimental systems

n/a	Involved in the study	
☒	☐	Antibodies
☒	☐	Eukaryotic cell lines
☒	☐	Palaeontology and archaeology
☒	☐	Animals and other organisms
☒	☐	Clinical data
☒	☐	Dual use research of concern
☐	☒	Plants

Methods

n/a	Involved in the study	
☒	☐	ChIP-seq
☒	☐	Flow cytometry
☒	☐	MRI-based neuroimaging

Dual use research of concern

Policy information about dual use research of concern

Hazards

Could the accidental, deliberate or reckless misuse of agents or technologies generated in the work, or the application of information presented in the manuscript, pose a threat to:

No	Yes	
x		Public health
x		National security
x		Crops and/or livestock
x		Ecosystems
x		Any other significant area

Experiments of concern

Does the work involve any of these experiments of concern:

No	Yes	
x		Demonstrate how to render a vaccine ineffective
x		Confer resistance to therapeutically useful antibiotics or antiviral agents
x		Enhance the virulence of a pathogen or render a nonpathogen virulent
x		Increase transmissibility of a pathogen
x		Alter the host range of a pathogen
x		Enable evasion of diagnostic/detection modalities
x		Enable the weaponization of a biological agent or toxin
x		Any other potentially harmful combination of experiments and agents

Plants

Seed stocks	No seed stocks were involved. Sampling of young leaf material into dessicant was performed in the field, aiming for at least 10 plants per population, with each sampled plant a minimum of 2 meters from any other. All locations are given in Dataset S1. Collection dates and locations are detailed for all samples in the ENA archive at EMBL-EBI under accession number PRJEB66308 (https://www.ebi.ac.uk/ena/browser/view/PRJEB66308).
Novel plant genotypes	All plant materials were naturally collected.
Authentication	No novel genotypes were produced or authenticated.
